# 

**Published:** 1903-02-07

**Authors:** 


### NOTICE TO CORRESPONDENTS.

All MSS., Letters, Books for Review, and other matters intended for the Editor should be addressed The Editor, "The Hospital," The Hospital Building, 28 & 29 Southampton Street, Strand, W.C.

The Editor cannot undertake to return rejected MS., even when accompanied by stamped directed envelope.

**Notice to Advertisers and Subscribers.**

All Advertisements, Orders for Copies of The Hospital, and Business Communications should be addressed to **THE MANAGER, (Not to the Editor)**, The Hospital, 28 & 29 Southampton Street, Strand, London, W.C.

The Cost of Subscription, post free, is as follows.—Per week, 2½d.; per quarter, 2s. 8d.; per half-year, 5s. 3d.; per annum, 10s. 6d. Foreign:—Per annum, 15s. 2d., payable, in all cases, in advance.

**NOTICE.—Vol. XXXII. of "The Hospital" (April to September 1902) in handsome cloth binding is now on sale at the office of this Journal, price, 7s. 6d. Cases for binding the Numbers contained in this Volume 1s. 6d. Index to Vol. XXXII., 2½d., post free.**

**The Monthly Part for February is now ready. Price 6d., by post 8d.**

### BOOKS RECEIVED.

The Scientific Press.

"A Practical Handbook of Midwifery." By Francis W. Nicol Haultain, M.D., F.R.C.P.E. Second edition, entirely revised (illustrated).

Baillière, Tindall and Cox.

"Pulmonary Consumption." By Arthur Latham, M.A., M.D.

John Bale, Sons, and Danielsson, Ltd.

"The Surgical Treatment of Ulcer of the Stomach." By C. Mansell Moullin, M.D., F.R.C.S.

inquiry is subsequently made by the house committee, and if deemed necessary other investigations are instituted in the case of all in-patients, and by these means the risk of abuse of the Charity is greatly diminished, and the Board is satisfied that little abuse, if any, prevails." I entirely acquit Mr. Bentham of any intention to misrepresent the case, but that he has done so is abundantly evident from the report.

### Scraps and Gleanings.

In the Chancery Division, on January 23rd, the case of Apollinaris Company, Limited *v.* E. R. Shaw, Limited, which related to an alleged infringement of the plaintiffs' trade mark for mineral waters, was settled. The defendants undertook not in any way to infringe the Apollinaris trade mark and to deliver up the labels complained of in their possession, and it was arranged that all proceedings in the action should be stayed. Mr. Justice Buckley made an order as agreed.

The Playgoer's Club are repeating their kindly and generous scheme of last year by arranging for 16,000 children of poor parents to witness a pantomime. The first of these pantomime treats took place on January 24th, when 3,200 children from the Board Schools and institutions of Hoxton and the neighbouring districts visited the Britannia Theatre, where a special performance of "King Krooked" was given for their benefit. The arrangements for seating this large party were excellently carried out, and the children had a thoroughly good time. Each one of them was presented with a bag containing a meat pie, a piece of cake, two ounces of sweets, and an orange. During the next few weeks the remaining 12,000 odd children will be taken to the West London, the Surrey, the Pavilion, the Kennington, and other theatres. It is understood that those children who have never seen a pantomime are primarily selected.

### The Student of Medicine.

### Appointments.

**J. Gray Clegg,** M.D., B.S., F.R.C.S., appointed Honorary Surgeon of the Manchester Royal Eye Hospital. **W. McAdam Eccles,** M.S.Lond., F.R.C.S.Eng., appointed Assistant Surgeon to St. Bartholomew's Hospital, London. **William Alexander Fleming,** M.B., M.S., appointed Public Vaccinator for Catlin's District, New Zealand. **George Alfred Harrison,** M.R.C.S., appointed Public Vaccinator for the Eltham District, New Zealand. **W. A. Harrison,** M.B., C.M.Edin., appointed District Medical Officer at York, West Australia. **John Hay,** M.D.Vict., M.R.C.S., L.R.C.P., appointed Honorary Pathologist to the David Lewis Northern Hospital, Liverpool. **H. C. Highet,** M.D., C.M., D.P.H.Lond., appointed Medical Adviser to his Siamese Majesty's Customs Department, Bangkok, Siam. **Charles A. Hill,** M.B.Cantab., D.P.H.Vict., appointed Honorary Bacteriologist to the David Lewis Northern Hospital, Liverpool. **T. C. Litler Jones,** F.R.C.S.Eng., appointed Honorary Assistant Surgeon to the Liverpool Royal Infirmary. **J. M. Longford,** L.R.C.P., L.R.C.S.Irel., appointed Resident Medical Officer to the Stoke-upon-Trent Union Workhouse. **Alfred E. Mole,** M.B., C.M.Edin., appointed Medical Officer of Health to the Gower District Council. **Ayres Moore,** L.R.C.P., L.R.C.S.Irel., appointed District Medical Officer of the Lutterworth Union. **William Paynter Noall,** M.B., B.S.Lond., M.R.C.S.Eng., L.R.C.P.Lond., appointed House Surgeon to the Bradford Royal Infirmary. **Norman Rainir,** M.R.C.S., L.R.C.P., Captain I.M.S., appointed Clinical Assistant to the Chelsea Hospital for Women. **Douglas Seaton,** M.B., Ch.B.Vict., appointed Honorary Surgeon to the Leeds Public Dispensary. **Michael A. Teale,** M.A.Oxon., M.R.C.S., L.R.C.P. Lond., appointed Honorary Ophthalmic Surgeon to the Leeds Public Dispensary. **J. S. F. Weir,** M.B., B.S., R.U.I., appointed Assistant Medical Officer to the Hendon Asylum of the Central London Sick Asylum District.

**"The Hospital" Institutional Library.**

**"Hospitals and Asylums of the World." 4 Vols., with a Portfolio of Plans, complete £12 12s.**
**"Burdett's Hospitals and Charities, 1902." 5s.**
**"Cottage Hospitals, General, Fever, and Convalescent." 10s. 6d.**
**"Hospital Expenditure: The Commissariat." 2s. 6d.**
**"A Legal Handbook for the Use of Hospital Authorities." 2s. 6d.**
**"The Cottage Hospital Case Book or Register of Patients." 10s. and 12s. 6d.**
**"The Furnishing and Appliances of a Cottage Hospital." 6d.**
**All these are published by the Scientific Press, Ltd., and may be obtained through any bookseller, or direct from the publishers, 28 and 29 Southampton Street, Strand, London, W.C.**

### A UNIQUE OPPORTUNITY

FOR ALL INTERESTED IN, OR CONNECTED WITH, HOSPITALS AND INSTITUTIONS.

The result of FOURTEEN YEARS' strenuous work and an expenditure of the enormous sum of **£6,500,**

**for £12 12s.**

on **The Times** plan

OF

**TWELVE Monthly Payments of ONE GUINEA each.**

### HOSPITALS & ASYLUMS OF THE WORLD:

**Their Origin, History, Construction, Administration, Management, and Legislation, with Plans of the chief Medical Institutions accurately drawn to a uniform scale.**

### By SIR HENRY BURDETT, K.C.B.

In Four Volumes, with separate Portfolio (20 in. by 14 in.) containing some hundreds of Plans. Royal 8vo. cloth extra, top gilt, **£12 12s.** complete.

Vols. I. & II. { Asylums and Asylum Construction | Vols. III. & IV. { Hospitals and Hospital Construction.

This valuable Encyclopædic Work contains exhaustive information on every phase of Hospital Construction, Organisation, and Management, **whilst special chapters are devoted to the History of Nursing and the Training of Nurses and Asylum Attendants.**

It is recorded that the late Mr. W. E. GLADSTONE, having read the whole of the four volumes carefully, declared it to be one of the **most interesting and remarkable works** it had ever been his good fortune to peruse.

Persons desiring to avail themselves of this Special Offer should send immediately for Order Form and Complete Prospectus, **as only a Few Copies of the Book remain Unsold,** and this offer will be withdrawn directly these are disposed of.

---

**Publishers: THE SCIENTIFIC PRESS, LTD., 28 & 29 Southampton Street, Strand, London, W.C.**

